# Haemoglobin (Hb) AE Bart’s Disease in a Young Patient: A Case Report

**DOI:** 10.7759/cureus.76277

**Published:** 2024-12-23

**Authors:** Sumaiyah Adzahar, Adibah Daud, Sharifah Nany Rahayu Karmilla Syed Hassan, Mohammad Hudzaifah Nordin, Nabilah Rameli, Nurul Asyikin Nizam Akbar, Razan Hayati Zulkeflee

**Affiliations:** 1 Department of Pathology and Medical Laboratory, Faculty of Medicine, Universiti Sultan Zainal Abidin (UniSZA), Kuala Terengganu, MYS; 2 Department of Pathology, Faculty of Medicine, Universiti Sultan Zainal Abidin (UniSZA), Kuala Terengganu, MYS; 3 Department of Ophthalmology, Faculty of Medicine, Universiti Sultan Zainal Abidin (UniSZA), Kuala Terengganu, MYS; 4 Department of Haematopathology, Hospital Dungun, Kuala Dungun, MYS; 5 Department of Haematology, School of Medical Sciences, Universiti Sains Malaysia, Kota Bharu, MYS

**Keywords:** hb ae bart’s disease, hb constant spring, hb h disease, sea deletion, thalassemia

## Abstract

Haemoglobin (Hb) AE Bart’s disease is a rare form of thalassemia that results from the co-inheritance of Hb E and alpha thalassemia, typically with Hb H disease. The clinical severity can vary depending on the underlying genetic mutations, particularly in the presence of Hb Constant Spring (Hb CS), which is a highly unstable form of alpha thalassemia. Understanding the genetic basis and haematological profiles of Hb AE Bart’s disease is crucial for proper diagnosis and management. We report the case of a nine-year-old Malay boy presenting with severe hypochromic microcytic anemia, jaundice, and hepatosplenomegaly. Haemoglobin electrophoresis findings consistent with Hb H Constant Spring disease co-inherited with Hb E. Molecular genetic testing confirmed compound heterozygosity for the South East Asian (SEA) deletion and Hb CS mutation along with heterozygous Hb E. This case highlights the importance of considering complex haemoglobinopathies such as Hb AE Bart’s disease in patients presenting with anemia, especially in regions with a high prevalence of thalassemia. Early diagnosis through a combination of Hb electrophoresis and molecular genetic testing is essential for proper management and genetic counseling to prevent long-term complications.

## Introduction

Thalassemia is a genetic disorder of the globin chains that make up haemoglobin (Hb) [1,2]. Alpha thalassemia results from deletion or mutations in the alpha gene, leading to decreased production of alpha globin chains. The severity of the disease is determined by the number of functional alpha globin genes still intact when both alleles are compromised [3].

The South East Asian (SEA) deletion is a common cause of alpha zero thalassemia, particularly in Southeast Asian populations [3,4]. This deletion removes both alpha globin genes on the same chromosome 16, resulting in a complete lack of alpha globin production. Conversely, Hb Constant Spring (Hb CS) is a non-deletional alpha thalassemia variant with an elongated and unstable alpha globin chain. This variant can exacerbate the clinical severity of thalassemia when combined with other alpha-thalassemia mutations [4]. Compound heterozygosity for SEA deletion and Hb CS leads to Hb H disease due to a significant reduction in alpha globin chain production [5].

Furthermore, Hb E is a common structural Hb variant in Southeast Asia [5], when interacting with Hb H disease, resulting in Hb AE Bart’s disease. Here we report a case of Hb AE Bart’s disease in a nine-year-old boy who presented with severe anaemia, jaundice, and splenomegaly. Understanding the haematological profiles and genetic implications of Hb AE Bart’s disease is crucial for accurate diagnosis, genetic counselling, and appropriate management of individuals with these conditions.

## Case presentation

A nine-year-old Malay boy presented to our clinic with a two-day history of fever, chronic fatigue, pallor, and jaundice. Physical examination revealed mild pallor and jaundice, along with palpable hepatosplenomegaly. His mother mentioned that he had occasionally looked jaundiced since he was one year old but had not sought medical attention as he remained active. There was a significant family history of thalassemia in the family; both parents had been diagnosed with thalassemia carrier, and the patient's brother was known to have alpha thalassemia. However, there was no family history of recurrent blood transfusions.

**Figure 1:**
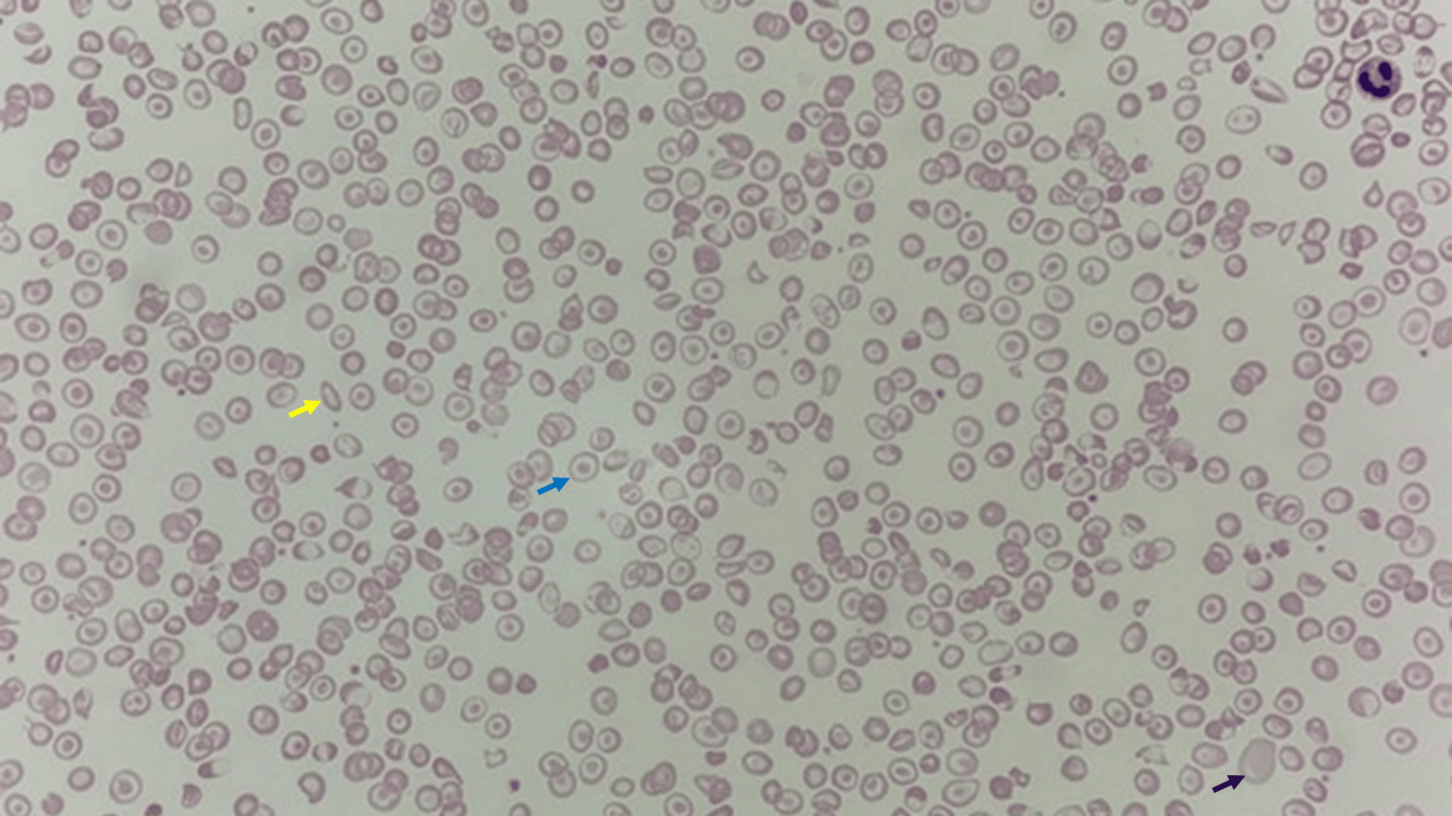
Peripheral blood examination showed anisopoikilocytosis with microcytic hypochromic red cells, numerous target cells (blue arrow), elliptocytes (yellow arrow) and polychromatic cells (black arrow); Magnification x40, Wright stain

**Figure 2:**
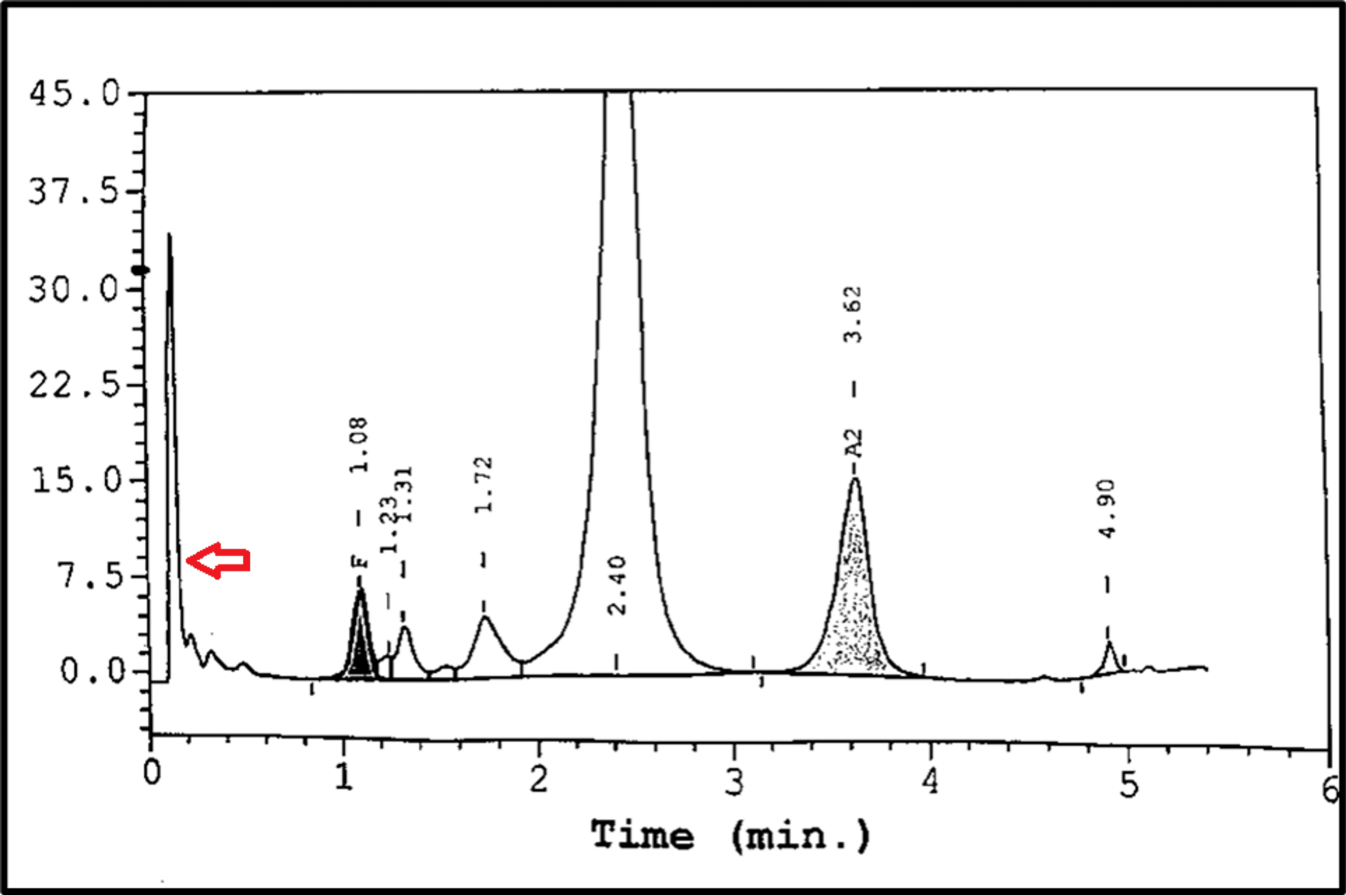
HPLC reveals the presence of a pre-run peak (red arrow), with prominent peaks observed in the F, A2, and C windows HPLC: high-performance liquid chromatography

A complete blood count (CBC) showed severe anaemia with a Hb level of 5.5 g/dL, a low mean corpuscular volume (MCV) of 46 fL, a low mean corpuscular Hb (MCH) of 14.2 pg, and a high red cell distribution width (RDW) of 20.9%. Peripheral blood smear revealed anisopoikolocytosis with microcytic hypochromic red cells, numerous target cells, elliptocytes, and polychromatic cells (Figure 1). Hb analysis with capillary electrophoresis showed Hb A 78.8%, Hb F 4.8%, Hb A2 2.4%, Hb E 11.1%, Hb Bart 1.3%, Hb H 0.8%, and Hb C 0.8%, which was consistent with Hb E trait co-inherited with Hb H Constant Spring. High-performance liquid chromatography (HPLC) revealed the presence of a pre-run peak indicating Hb H, with some peaks observed in the F, A2, and C windows (Figure 2).

**Figure 3:**
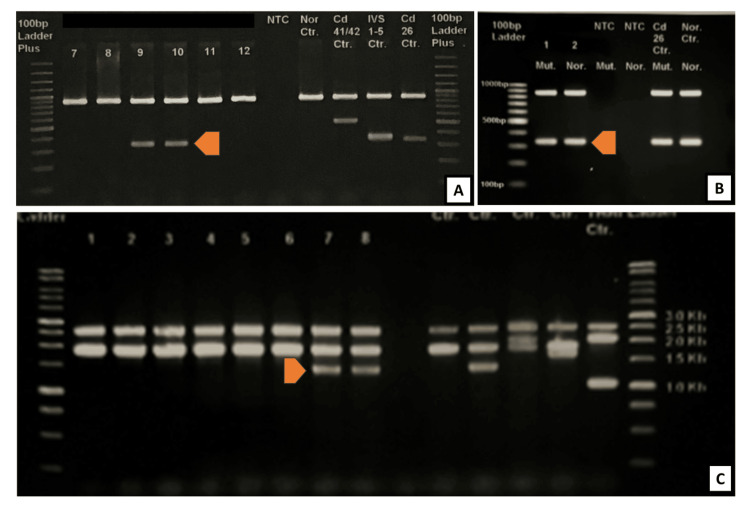
Molecular studies for alpha and beta thalassemia (A) Beta multiplex ARM molecular analysis showed a positive result for Codon 26 at an amplicon size of 301 bp for both lane 9 and 10; (B) Zygosity testing revealed the presence of both mutant and normal alleles for both lane 1 and 2, indicating that the patient is heterozygous for Codon 26; (C) Lanes 7 and 8 displayed a heterozygous SEA deletion detected at 1349 bp using the Gap-PCR method *The figure for alpha molecular studies (ARMS method) is not available ARM: amplification refractory mutation; PCR: polymerase chain reaction; SEA: South East Asian

Molecular testing for alpha thalassemia (both deletion and non-deletion) was done for the patient and revealed compound heterozygosity for alpha-zero thalassemia (−−SEA) and Hb CS, consistent with Hb H CS. Additionally, beta molecular studies confirmed heterozygosity for Hb E (Codon 26, G>A) (Figure 3). The total bilirubin was elevated at 113.1 µmol/L, with a predominance of indirect bilirubin (67.2 µmol/L), reflecting ongoing haemolysis. The laboratory investigations are summarized in Table 1. The patient was treated with packed red blood cell transfusions and folic acid supplementation for anaemia. Regular follow-ups were arranged to monitor Hb levels, liver function, and iron status.

**Table 1:** Laboratory investigations MCV: mean corpuscular volume; MCH: mean corpuscular haemoglobin; Hb: haemoglobin

Test	Parameters	Result	Reference ranges
Complete blood count	Haemoglobin	5.5	11.5-15.5 g/dL
	MCV	46.0	80-100 fL
	MCH	14.2	27-34 pg
	RBC	3.87	4.0-5.2 x 10^12^/L
	RDW	20.9	11.0-16.0 %
	Platelet	150	170-450 x 10^9^/L
	WBC	10.5	4 – 10 x10^9 ^/L
	Reticulocytes	1.5	0.1-2.0 %
Liver Function Test	Total Bilirubin	113.1	5-21umol/L
	Indirect Bilirubin	67.2	0-12 umol/L
	Direct Bilirubin	45.9	0-3.4 umol/L
Haemoglobin analysis (capillary electrophoresis)	Hb A	78.8 %	>90%
	Hb A2	2.4 %	-
	Hb F	4.8 %	<1%
	Hb E	11.1 %	-
	Hb CS	0.8 %	-
	Hb Bart	1.3 %	-
	Hb H	0.8 %	-

## Discussion

**Figure 4:**
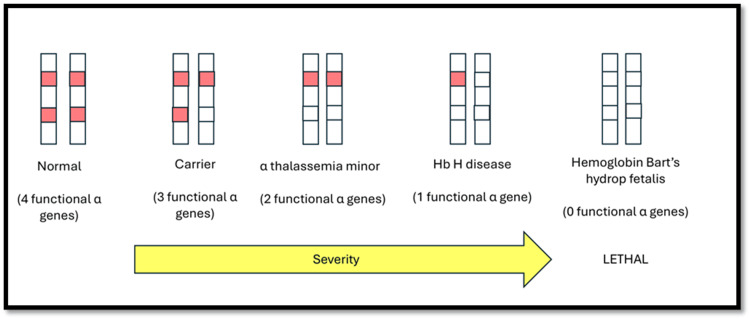
Genetics and clinical Implications of alpha thalassemia Image Credit: Sumaiyah Adzahar

Hb H disease is a type of alpha thalassemia that occurs due to the deletion or inactivation of three out of the four alpha globin genes (-/- or -/αα) (Figure 4). It is most commonly found in Southeast Asia, the Mediterranean, and the Middle East regions. The deficiency in alpha globin chains leads to the formation of beta-globin tetramers, which are unstable and precipitate within red blood cells, causing hemolysis [3].

Hb E disease results from a codon 26 (G>A) mutation in the beta-globin gene and, when combined with Hb H disease, leads to Hb AE Bart’s disease [6,7]. This case of a nine-year-old boy with Hb AE Bart’s disease underscores the genetic diversity of thalassemia in this region and highlights the importance of considering multiple alpha-thalassemia mutations when diagnosing hemoglobinopathies. The SEA deletion in this case, which eliminates two alpha-globin genes on one chromosome, combined with the Hb CS mutation, exacerbates the clinical severity due to an even further reduction in functional alpha-globin chains [3].

Clinically, Hb AE Bart’s disease mimics Hb H disease, presenting with moderate to severe anaemia, jaundice, and hepatosplenomegaly [8]. Furthermore, a study in central Thailand found that 86% of patients with non-deletional AE Bart's disease required more frequent transfusions, compared to only 12.5% of patients with deletional AE Bart’s disease [9].

The diagnostic workup, in this case, was guided by a combination of haematological findings and genetic testing, which is essential in cases of mixed haemoglobinopathies. Hb electrophoresis revealed reduced Hb A and Hb E, with small amounts of Hb Bart’s and Hb H. The lower affinity of alpha globin chains for beta E-globin chains compared to beta A-globin chains leads to decreased Hb E levels (13-15%), differentiating it from classic Hb H disease [9]. While electrophoresis is a useful initial tool, its limitations in detecting unstable Hbs, such as Hb CS, were evident in this case. The rapid degradation of Hb CS during storage at 4°C can lead to misinterpretation of electrophoresis results, as previously reported by Thonglairuam et al. [7]. Therefore, DNA analysis was crucial in confirming the compound heterozygosity for the SEA deletion and Hb CS mutation. The potential for diagnostic inaccuracies in these cases highlights the importance of molecular testing, particularly when electrophoresis alone may not provide definitive results.

Management of Hb AE Bart’s disease follows the principles applied in Hb H disease, focusing on symptomatic treatment and prevention of complications. In this case, the patient was managed with regular blood transfusions, folic acid supplementation, and iron chelation therapy [10]. As with all chronic hemolytic anaemias, iron overload is a significant concern in regularly transfused patients. Therefore, monitoring iron levels and liver function is essential to prevent hemosiderosis and organ damage. Furthermore, genetic counselling is critical for families with a history of thalassemia and hemoglobinopathies. In this case, both parents were carriers, emphasizing the importance of carrier screening in at-risk populations.

## Conclusions

This case provides valuable insight into the interaction between Hb E, Hb CS, and SEA alpha thalassemia in a Southeast Asian context. While Hb AE Bart’s disease is rare, it serves as a reminder of the genetic diversity seen in hemoglobinopathies. The incorporation of molecular testing into routine diagnostic pathways can enhance diagnostic accuracy, particularly in regions with a high prevalence of thalassemia mutations. Early diagnosis allows for appropriate management and genetic counseling to prevent future complications.
